# Scanning electron microscopic observation of the in vitro cultured protozoan, *Perkinsus olseni*, isolated from the Manila clam, *Ruditapes philippinarum*

**DOI:** 10.1186/s12866-020-01926-0

**Published:** 2020-08-03

**Authors:** Dinesh Gajamange, Seung-Hyeon Kim, Kwang-Sik Choi, Carlos Azevedo, Kyung-Il Park

**Affiliations:** 1 Department of Aquatic Life Medicine, College of Ocean Science and Technology, Kunsan National University, 558 Daehakno, Gunsan, 54150 Republic of Korea; 2 Present address: The Open University of Sri Lanka, Regional Centre, Matara, Sri Lanka; 3 School of Marine Biomedical Sciences, College of Ocean Sciences, Jeju National University, 102 Jejudaehakno, Jeju, 63243 Republic of Korea; 4 Laboratory of Cell Biology, Institute of Biomedical Sciences, University of Porto, Porto, Portugal

**Keywords:** *Perkinsus olseni*, life cycle, Manila clam, Morphology, SEM

## Abstract

**Background:**

Perkinsosis is a major disease affecting the commercially important marine mollusk *Ruditapes philippinarum* (Manila clam) in Asian waters. In this study, we investigated the morphological characteristics of *Perkinsus olseni*, the causative agent of perkinsosis, cultured under laboratory conditions at different stages of its life cycle using a scanning electron microscope (SEM).

**Results:**

The prezoosporangia formed after induction with Ray’s fluid thioglycollate medium (RFTM) developed into zoosporangia. During this process, a discharge tube formed a porous sponge-like structure that detached before the zoospores were released; thus, this organelle operated as a bung. Liberated zoospores gradually transformed into immature trophozoites, during which detachment of the anterior flagella occurred, but the loss of the posterior flagella was not clearly observed in the present study. Mature trophozoites underwent schizogony by cleaving the cell forming some merozoites in schizonts, which were released by the rupturing of the cellular membrane of the schizont within a few days.

**Conclusions:**

Our morphological and ultrastructural studies contribute new information on the life cycle and propagation of *P. olseni*.

## Background

*Perkinsus olseni* is a parasitic protozoan and is the causative agent of perkinsosis in the Manila clam (*Ruditapes philippinarum*), which is found in tidal flats and sandy beaches along the coasts of Asia and Europe [1–4]. The Manila clam found in Korean waters is an important edible shellfish of the mariculture industry; however, recurrent mass mortalities of this species have significantly impacted mariculture production [5]. Histopathological studies have reported that severe inflammatory reactions can occur in the connective tissue of the gonad, gills, mantle, stomach, and digestive gland due to severe *P. olseni* infections that could have deleterious effects on reproductive efforts and food digestion [6–8]. It was reported that the recent summer mortality of Manila clams on the west coast of Korea was caused by the combined effects of *P. olseni* infection and thermal stress [9]. In Japan, the negative effects of *P. olseni* on the survival of wild clam populations in Ariake Bay were also reported [10].

Since the first occurrence of *P. olseni* in Manila clams in Asia, described in the late 1990s by molecular studies [2, 11, 12], other parasites, such as *P. honshuensis* were associated with Manila clams*.* Of note, this new parasite species has been described in Manila clams from Japan and in *R. variegatus* collected on Jeju Island, on the southern coast of Korea, after a nation-wide survey of *P. honshuensis*: *P. honshuensis* specific primers were used to screen clams from 23 different locations [13–15]. Thus, the distribution of *P. honshuensis* appears to be restricted to Jeju Island (with respect to Korea) at the present.

The life cycle of *Perkinsus* spp. involves several life stages, namely, the trophozoite, prezoosporangium, zoosporangium, and zoospore stages [16–18]. The trophozoite stage is the vegetative proliferation stage that occurs in the host tissue; of note, and it has the characteristic signet-ring structure. This stage is the parasitic stage causing some pathological effects, including reduced host immunity, increased hemocyte infiltration, and the formation of nodular lesions in the host [19, 20]. The prezoosporangium stage is considered to be the dormant stage in the life cycle of *Perkinsus* spp. This stage allows the organism to endure unfavorable conditions [21, 22]. When trophozoites are exposed to anaerobic conditions, they grow larger and transform into thick-walled cells. This phenomenon was confirmed by incubating host tissue infected with *Perkinsus* spp. in liquid RFTM [1, 23]. The enlargement of trophozoites has also been observed in moribund hosts [24–26]. When isolated prezoosporangia are introduced to seawater or culture medium, they transform into zoosporangia and have multiple cell stages through progressive karyokinesis and cytokinesis [16]. Finally, the zoosporangia release motile zoospores into the medium via the discharge tube [16]. Zoospores gradually lose their zoospore characteristics, such as flagella and body shape, and transform into immotile trophozoites [27]. Subsequently, they develop into schizonts and finally release multiple daughter cells called merozoites [28].

Thus, the life cycle of the pathogen is well documented. However, these previous studies mainly used light microscopic analyses rather than electron microscopic observations; consequently, the descriptions of the morphological characteristics of *Perkinsus* spp. are limited. Therefore, here, we performed an in-depth analysis of the surface ultrastructure of *P. olseni* at each of its life stages using a light microscope (LM) and SEM. Our study is expected to provide new insights into the life cycle and transmission of the pathogen.

## Results

### Zoosporulation of in vitro cultured *P. olseni*

**Fig. 1:**
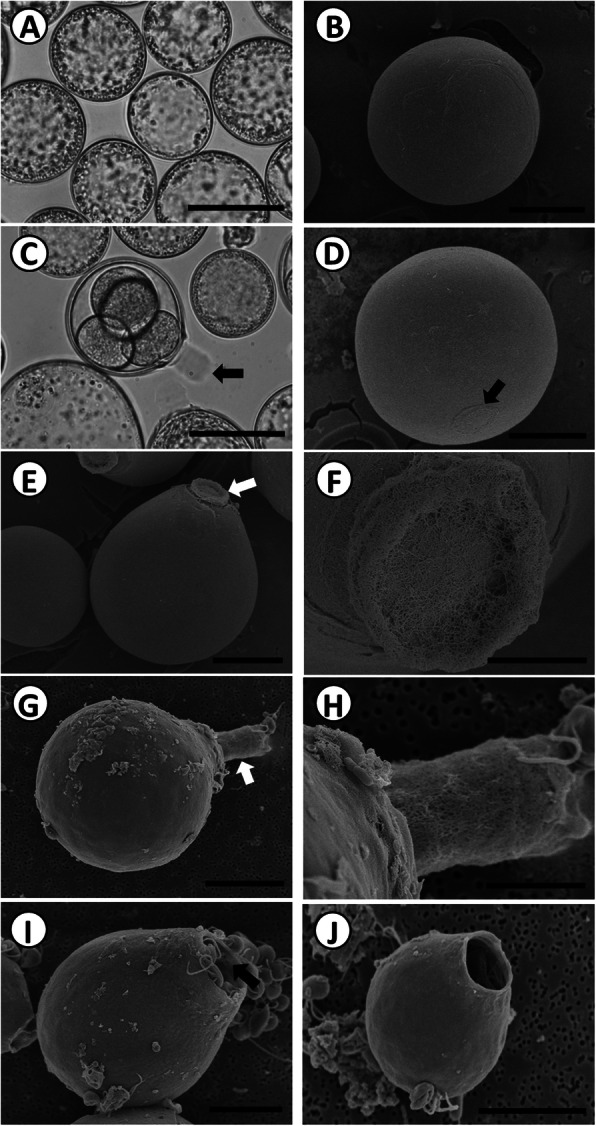
External structures of *P. olseni* during zoosporulation, observed under light microscopy (**a** and **c**) and FE-SEM (**b**, **d**, **e**, **f**, **g**, **h**, **i**, and **j**). **a**. Prezoosporangia on the second day in seawater taken under a light microscope; scale bar = 50 μm. **b**. Prezoosporangia on the second day in seawater taken under SEM; scale bar = 25 μm. **c**. Zoosporangia on the third day and a 4-cell stage of zoosporangium with the discharge tube (arrow); scale bar = 50 μm. **d**. Initiation of discharge tube formation (arrow) on the third day; scale bar = 25 μm. **e**. Development of the discharge tube (arrow) on the third day; scale bar = 20 μm. **f**. Higher magnification of the discharge tube showing the closed membrane mesh-like structure; scale bar = 5 μm. **g**. Fully developed discharge tube on the fourth day; scale bar = 15 μm. **h**. Magnified view of the discharge tube; the tube looks like a porous sponge; scale bar = 5 μm. **i**. Disintegrated discharge tube and release of zoospores from the zoosporangium on the fifth day; scale bar = 10 μm. **j**. Empty zoosporangium after complete discharge; scale bar = 10 μm

The different morphological life stages in the progression to zoosporulation in vitro in culture media are presented in Fig. 1. On the second day of in vitro culture, prezoosporangia were spherical, as observed under LM (Fig. 1a), which was consistent with the electron microscope observation showing that prezoosporangia have a smooth body wall (Fig. 1b). They ranged from 28.1 to 105.1 μm in diameter (mean, 61.1 ± 16.8 μm; *n* = 100). On the third day, various phases of cell division were observed with LM. SEM showed that 7% of the cells showed the development of a discharge tube that continuously elongated, although it remained plugged until the apparatus had completely formed. LM showed that successive bipartition took place through karyo- and cytokinesis, giving rise to zoosporangia containing different numbers of cells inside (Fig. 1c). The external growth of the discharge apparatus was shown by SEM (Fig. 1d). When elongation was initiated (Fig. 1e), it had a mesh-like structure (Fig. 1f). On the fourth day, 70% of the zoosporangia had a discharge tube. The discharge tube developed into a rod shape that had a porous sponge-like structure (Fig. 1g-h). The lengths of the discharge tube ranged from 9.8 to 18.6 μm (14.2 ± 2.6 μm, *n* = 15). The length-to-diameter (zoosporangia body) ratio was 0.4 ± 0.1 μm (n = 15). Twenty-five percent of the cells became zoosporangia, with approximately 100 zoospores being closely packed in the lumen of the zoosporangium, which ranged from 28.4 to 90.7 μm in diameter (53.9 ± 13.5 μm, *n* = 100). On the fifth day, most gravid zoosporangia completed zoosporulation, and the motile zoospores had been discharged into the culture medium. SEM clearly showed that the discharge tube disintegrated, leaving an opening from which the motile zoospores were emerging (Fig. 1i) and had emerged (Fig. 1j).

**Fig. 2:**
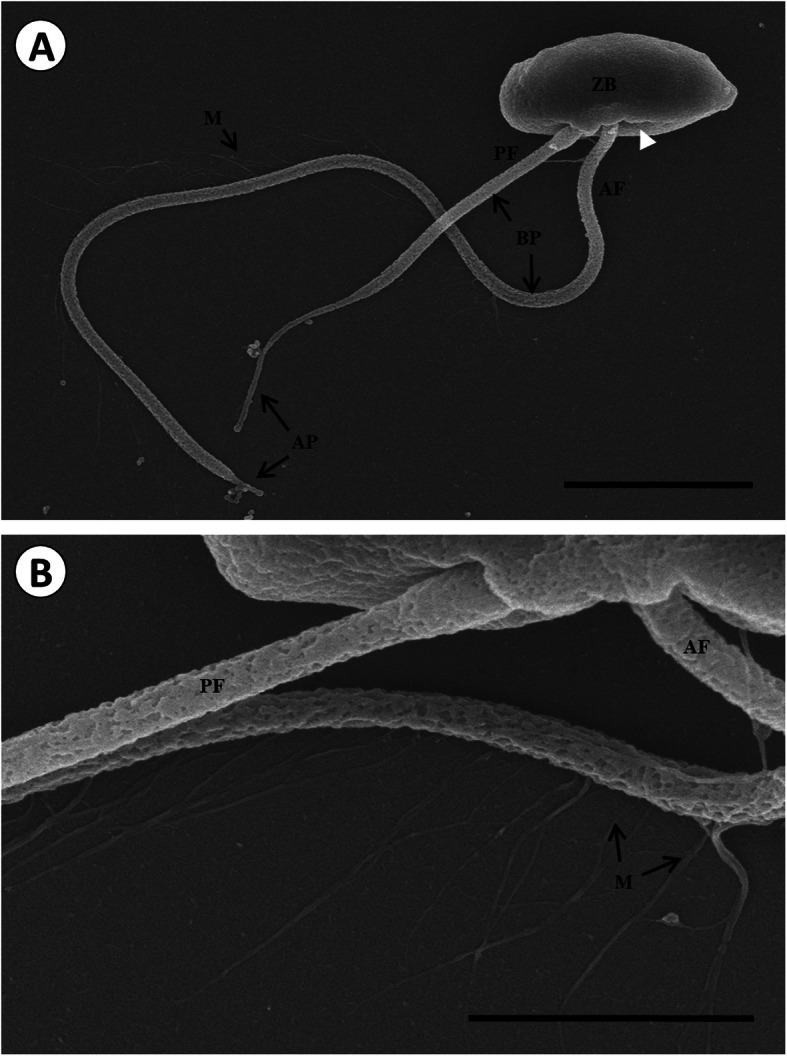
Scanning electron micrograph of *P. olseni* zoospore (**a**) and tinsel (mastigonemes, M) (**b**). A zoospore with the anterior flagellum (AF) and posterior flagellum (PF). The flagellum is rooted in the zoospore body (ZB). The flagellum is divided into two parts; the basal portion (BP) and an apical portion (AP). Unilateral array of tinsels on the anterior flagellum (M). Scale bar = 2 μm (**a**), Scale bar = 1 μm (**b**)

The released zoospores were highly motile, exhibiting circular and erratic movements. They were ellipsoidal; their dorsal surface was slightly convex, while their ventral surface was flattened (Fig. 2a). Their body dimensions were 3.7 ± 0.4 μm × 2.0 ± 0.2 μm (*n* = 60). The anterior flagellum was crooked, while the posterior one was slightly straight, with a rough surface. The anterior flagellum was 14.0 ± 1.0 μm long (*n* = 50) while the posterior flagella was shorter (7.0 ± 0.8 μm) (*n* = 50). The distal end of the anterior flagellum tapered into a short narrow tip (apical portion, AP); however, in the case of the posterior flagellum, the distal end started to taper to about two-thirds of the length of the posterior flagella and ended with a narrow tip. The anterior flagellum had a clear unilateral array of tinsel, distributed in-between, approximately 1 μm from the flagellum base up to the front of the AP (Fig. 2b). The tinsels were 1.3 ± 2.9 μm long and appeared at 0.3 ± 0.1-μm-long intervals,

### Transformation of zoospores to trophozoites

**Fig. 3:**
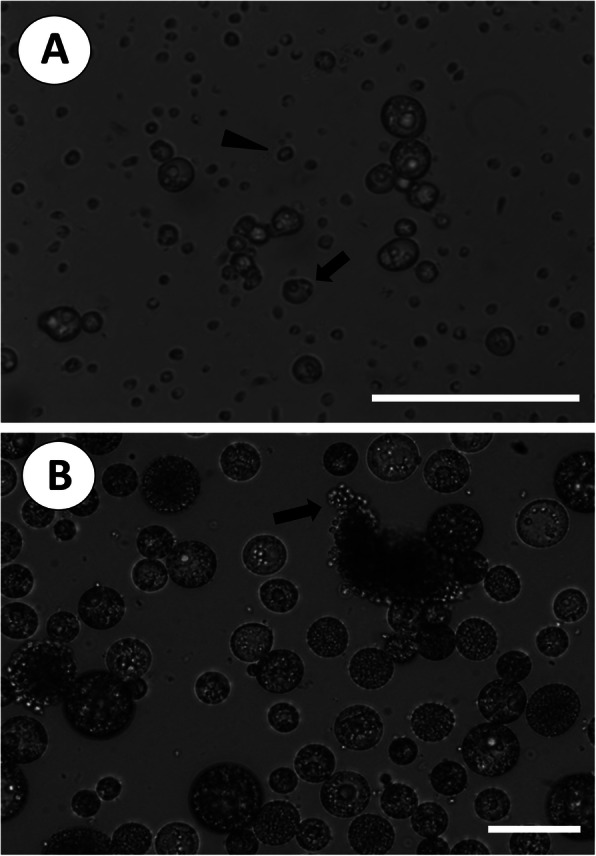
Microscopic images of *P. olseni* zoospores transforming into trophozoites and schizogony observed under a light microscope. **a**. Zoospores (arrowhead) released to the medium and transformed into trophozoites (arrow) on the third day; scale bar = 50 μm. **b**. Clusters of sibling merozoites (arrow) released from a schizont on the fifteenth day; scale bar = 20 μm

**Fig. 4:**
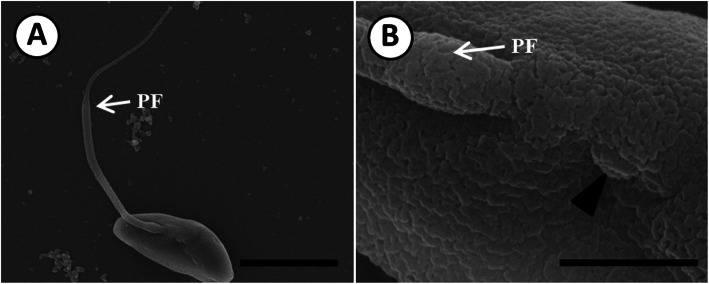
Scanning electron micrograph showing the deflagellation of *P. olseni* zoospores. **a**. Deflagellation of the anterior flagellum and the remaining posterior flagellum (PF); scale bar = 2.5 μm. **b**. The vestige of deflagellation (arrowhead) and the remaining posterior flagellum (PF); scale bar = 0.5 μm

The light microscopy and SEM images depicting zoospores transforming into trophozoites, are shown in Figs. 3 and 4, respectively. On the third day following discharge, most zoospores exhibited high motility, but some zoospores transformed into non-motile trophozoites and grew larger (Fig. 3a). During the transformation from zoospores to trophozoites, SEM showed that the anterior flagellum was lost first, while the posterior flagellum remained (Fig. 4a). At this stage, zoospores showed weak movement. Deflagellation took place at the junction between the basal part of the anterior flagellum and the body surface (Fig. 4b). Several detached anterior flagella were observed on the bottom of the culture well plate. However, the detachment of posterior flagella was not observed. The trophozoites exhibited a spherical or irregular shape on the fifth day (Fig. 3a), followed by the appearance of trophozoites containing a typical signet-ring structure with an uncommonly large vacuole during the seventh to eighth days. During the transformation period, the length of the zoospore gradually decreased from 3.7 μm on the first day to 3.3 μm on the twelfth day. The diameter of trophozoites increased from 4.8 μm on the third day to 31.5 μm on the fifteenth day, as the transformation rate increased (Table 1). It took 15 days for 87% of zoospores to transform into trophozoites.

**Table 1:** Transformation rate and dimensions of *P. olseni* trophozoites during transformation in the culture medium

Days after zoospores were released to the medium	Transformation rate (%)	Size (μm) ± SD	N
3	3	4.8 ± 1.2	40
5	6	8.0 ± 3.2	100
7	29	11.4 ± 4.9	100
9	36	15.3 ± 7.1	100
12	66	21.2 ± 9.2	100
15	87	31.5 ± 15.5	100

*SD* standard deviation

### Schizogony

**Fig. 5:**
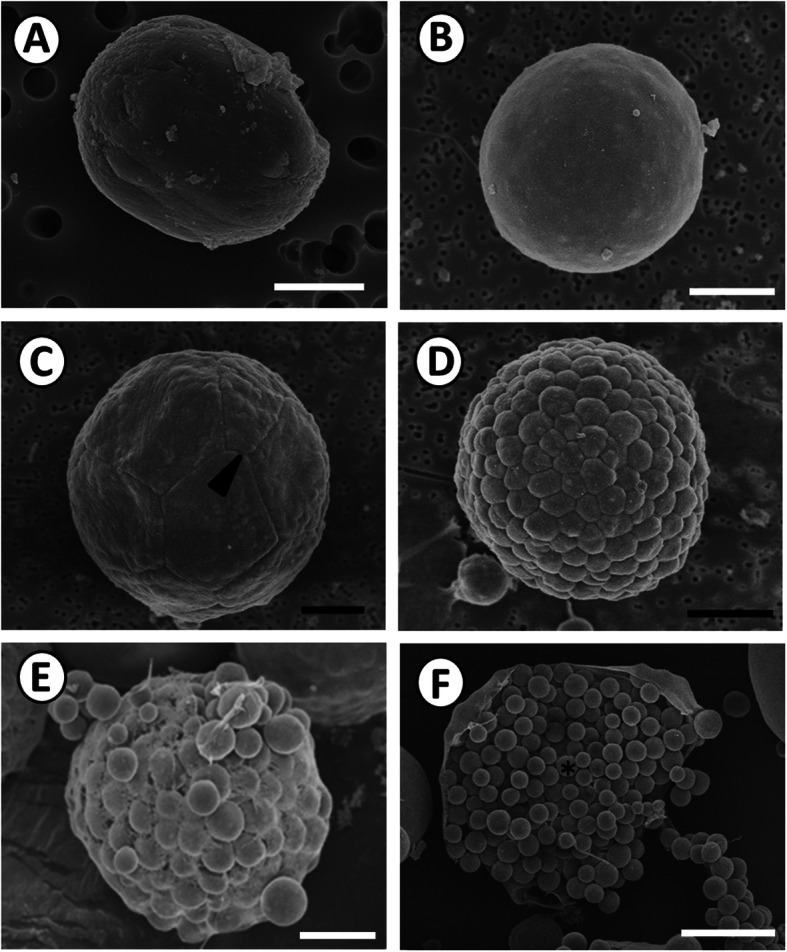
Scanning electron micrograph showing schizogony of *P. olseni*. **a**. Immature trophozoite with a spherical or irregular shape on the fifth day after the release of zoospores to the medium; scale bar = 1 μm. **b**. Mature trophozoite with a smooth surface on the ninth day; scale bar = 5 μm. **c**. Early schizont showing an early cleavage furrow (arrowhead) on the twelfth day; scale bar = 5 μm. **d**. Morula stage, showing approximately 100 irregularly shaped merozoites on the thirteenth day. Scale bar = 5 μm. **e**-**f**. Rupture of the schizont cellular membrane and release of spherically shaped merozoites on the fifteenth day; E: scale bar = 5 μm; F: scale bar = 15 μm. Asterisk indicates merozoites

The early-phase trophozoites were spherical or irregularly shaped (Fig. 5a). Their size gradually increased, and they had smooth surface structures once mature (Fig. 5b). Trophozoites enlarged and had cleavage furrows on the cell surface, indicating the early subdivision of the trophozoites, or the beginning of schizogony (Fig. 5c). As cleavage progressed, the number of daughter cells increased and progressed to the morula stage, with approximately 100 irregular-shaped merozoites being detected on the thirteenth day (Fig. 5d). When schizonts had fully developed, they released hundreds of merozoites, ranging in size from 1.4 to 7.8 μm (mean, 4.9 ± 1.4 μm; *n* = 100), following the abrasion or rupture of the schizont cellular membrane on the fifteenth day (Fig. 5e–f). Light microscopy also showed that schizonts had internal divisions and released merozoites (Fig. 3b).

### Identification of *P. olseni* by molecular diagnosis

The trophozoites obtained in the present study were confirmed to be *P. olseni* by PCR (data not shown).

## Discussion

The current study documented the morphological features of the life cycle of *P. olseni* isolated from Manila clams under in vitro conditions using SEM. In the life cycle of *P. olseni*, our first observation of the pathogen was the zoosporangia, and the most noticeable features of these cells were the morphology and function of the discharge tube. The term “discharge tube” was coined [29] for a hollow appendage that extruded from the wall of *P. marinus* zoosporangia and through which mature motile zoospores exited zoosporangia, after the dissolution of a plug that sealed a discharge pore in the thick zoosporangium wall. Similarly, *Parvilucifera prorocentri,* a tube-forming intracellular parasite of dinoflagellates, discharges zoospores through a discharge tube [30, 31]. However, unlike *P. prorocentri*, motile zoospores of *P. olseni* were released via a circular opening of the cell wall of the zoosporangium when the discharge tube disintegrated and separated from the zoosporangium, suggesting that the discharge tube functioned as a bung. The light microscopic analysis in the current study also showed the presence of an elongated rod-shaped organ (discharge tube) during the early phase of zoosporulation; however, when discharging zoospores, the elongated discharge tube disappeared, and zoospores were released directly from the zoosporangium. Several reports have included similar images, with no elongated discharging device at this stage [17, 32].

In the present study, motile zoospores started to transform into trophozoites on the third day of zoospore cultivation and lost their mobility, as observed under light microscopy. It was assumed that the reduced activity of zoospores was caused by a defect in flagella movement, suggesting the loss of flagella. The loss of flagella could be caused by flagellar detachment or resorption in response to various stimuli or the cell cycle [33]. Indeed, we were able to observe the vestige where the anterior flagellum was amputated from the body of the zoospore, and some detached anterior flagella were observed in the culture media by SEM. It was reported that deflagellation of *Chlamydomonas* spp., a green alga, occurs at the distal end of the flagellar transition zone, where axoneme microtubules terminate when they are exposed to various stresses, such as pH shock, heat, alcohol treatment, and mechanical shearing [34, 35]. During deflagellation, several proteins cause the fission of the flagellar membrane and the severing of the outer doublet microtubules; these proteins include centrin and katanin [36, 37]. In contrast, flagellar resorption is seen in nature in various groups of organisms, including *Hexamita*, amoebae, green algae, and fungi, during the transformation from flagellates into an amoeba, before cell division and encystment, or in response to stress [38–40]. However, in the present study, detachment of the anterior flagella was confirmed, but the posterior’s deflagellation remained doubtful. Thus, further TEM studies on flagellar disappearance in this pathogen must be conducted. As flagella are known to play important roles in cell motility, attachment, and host cell invasion [41], studies on the morphological features and function of *P. olseni* flagella will be useful for understanding the transmission of this pathogen.

Schizogony, asexual reproduction of protozoans by multiple fissions, is a typical method for the proliferation of the trophozoite phase of *Perkinsus* spp. Some studies described sibling daughter cells in vitro or in histological observation [17]. In our study, the schizogony of *P. olseni* from mature trophozoites to merozoites was shown in detail by SEM observation. In this process, each schizont in the early phase of schizogony consists of several long sides (polygonal shape) of merozoites. However, as schizogony progresses, the merozoites become smaller, and the number of sides of each polygon increases. Thus, the merozoites become circular. In the final phase of schizogony, hundreds of circular merozoites are released by rupture or abrasion of the schizont membrane. It is highly likely that schizont morphology and the number of merozoites of *P. olseni* induced by nutrient-enriched media in the present study might differ from those occurring in nature, as previously suggested [27]. However, our observation provides useful clues for understanding the multiple fission of *P. olseni*.

## Conclusion

In conclusion, we provided a detailed description of the external morphological features of *P. olseni* at all stages of its life cycle. Our study showed that the life cycle of *P. olseni* is generally similar to that previously reported. However, we also provide a detailed description of zoosporulation, transformation, and schizogony of the pathogen, which is expected to advance our understanding of the life cycle and transmission of the pathogen.

## Methods

### Clam sampling

Manila clams (*R. philippinarum*) were collected from intertidal flats of the Hongseong Region in the west coast of Korea, where heavy *P. olseni* infection has been recorded (unpublished data). Clams were collected in January 2011, brought to the laboratory, and reared in aquaria with recirculating seawater (temperature, 15–18 °C; salinity, 34 psu).

### Isolation of prezoosporangia

RFTM culture assay was conducted to obtain prezoosporangia of the pathogen. Gill tissues of six clams were inoculated in 40 mL of RFTM prepared according to the NOAA’s manual with slight modification [42]. Briefly, 29.3 g of Fluid thioglycollate medium (Sigma, USA) and 20.0 g of NaCl (Sigma, USA) was dissolved in H_2_O, boiled, autoclaved, then supplemented with 800 μL penicillin-streptomycin (10,000 units/mL; Gibco, USA) and 10 μl Nystatin-Chloramphenicol [0.1 g Nystatin (Sigma, USA) in 10 ml Chloramphenicol (Sigma, USA)] in a 50 mL conical tube. Tissues were incubated in the dark at 23 °C for 60 h and then rinsed twice with sterile artificial seawater (SASW; 35 psu, filtered through a 0.2-μm filter and autoclaved). The finely chopped tissues were suspended in 50-mL SASW, and prezoosporangia were separated from tissue particles by filtering the mixture through a sieve series of 100 μm, 50 μm, and 30 μm. The prezoosporangia retained on 50-μm and 30-μm sieves were thoroughly washed with SASW and mixed in a 15-mL conical tube. They were then further purified by centrifugation (73×*g*, 3 min).

### Induction of in vitro zoosporulation

Zoosporulation of *P. olseni* was induced by introducing purified prezoosporangia into the culture media. The culture medium was prepared, as reported by Gauthier and Vasta [30], for in vitro zoosporulation, with minor adjustments to the ingredients. In brief, Dulbecco’s Modified Eagle Medium (Sigma, USA) and Ham’s F-12 (Gibco, USA) were mixed in a 1:2 ratio and added to an oceanic natural sea salt mix (Oceanic Systems, USA) dissolved in autoclaved distilled water. The mixture was buffered with 5 mM HEPES (Gibco, USA) and 3.5 mM sodium bicarbonate (Sigma, USA) and supplemented with 5% fetal bovine serum (FBS, Sigma, USA). The medium was further sterilized by filtration through a 0.2-μm syringe filter and stored at 4 °C until use (salinity, 35 psu; pH 7.2–7.4). The culture medium was fortified with antibiotics, as mentioned previously. In vitro zoosporulation was performed in 12-well plates by suspending prezoosporangia in the culture medium at 15,000 cells/mL, followed by incubation at 26 °C.

### Transformation of zoospores to trophozoites and schizonts

Following the release of motile zoospores from mature zoosporangia, motile zoospores were aspirated with supernatant culture medium and transferred to a new 12-well culture plate. The transformation was observed at 26 °C. The percentage of zoospores that transformed to trophozoites was calculated by counting the number of trophozoites and zoospores in 10 random photographic images of culture plates that were taken every 24 h over 15 d using LM.

### LM and SEM

The in vitro cultured cells were observed under LM and SEM at the different life stages, including zoosporulation, transformation of trophozoites, and schizogony. For LM, a culture plate was observed using an inverted light microscope. Images were taken at different magnifications using a digital camera (Moiticam 2300, China) mounted on the microscope. For SEM, samples were obtained periodically after introducing prezoosporangia to the culture medium. Samples were fixed in 2.5% glutaraldehyde (Sigma, USA), incubated for 1 h at room temperature, and post-fixed for another 1 h at room temperature in 2% OsO_4_ (Fluka, electron microscopy grade, USA). After incubation, the fixed samples were transferred to a 0.45-μm polycarbonate membrane filter (Advantec MFS, USA) that was mounted on a 25-mm vacuum filter holder with a glass funnel and glass filter flask. The fixatives were rinsed by aspiration using 30- and 15-psu filtered artificial seawater, followed by rinsing with distilled water. Samples for SEM analysis of deflagellation of flagella were attached to a glass coverslip for 2 h. The other samples remained in the membrane filter. After washing, parasite cells were dehydrated with a graded series of ethanol by a routine method. The cells were critical point dried with CO_2_ using a critical point dryer (Leica EM CPD030, Germany). Dried filters and coverslips containing cells were coated with platinum and examined under SEM (Hitachi S4800, Japan) with an appropriate accelerated voltage. Cells were measured using the Motic Images Plus 2.0 mL multi-media software (Motic, China). All measurements were expressed as mean ± SD (standard deviation).

### Identification of *P. olseni*

Identification of *P. olseni* from the induced trophozoites was confirmed by PCR using *P. olseni*-specific primer pair developed from the non-transcribed spacer region (NTS) [43]. For this experiment, the genomic DNA was extracted by the AccuPrep DNA extraction kit (Bioneer, Korea). Forward and reverse primers were 5′-CATTATCGAGGTCTGTGGTGACG-3′ and 5′-ACGATAGGTCTGCTGAGCAAGC-3′, respectively. The PCR reactions were performed for extracted DNA using ExTaq polymerase (TaKaRa, Korea) by pre-incubation at 95 °C for 5 min, followed by 35 cycles of denaturation at 94 °C for 1 min, annealing at 55 °C for 1 min and extension 72 °C for 2 min, followed by final 5 min extension at 72 °C.

## Data Availability

The datasets used and/or analyzed during the current study are available from the corresponding author on reasonable request.

## Abbreviations

SEM: Scanning electron microscope
RFTM: Ray’s fluid thioglycollate medium
LM: Light microscope
NTS: Non-transcribed spacer

## References

[1] Choi KS, Park KI (1997). Report on the occurrence of *Perkinsus* sp. in the Manila clam *Ruditapes philippinarum* in Korea. Korean J. Aquacult..

[2] Hamaguchi MN, Suzuki N, Usuki H, Ishioka H (1998). *Perkinsus* protozoan infection in short-necked clam *Tapes* (*=Ruditapes*) *philippinarum* in Japan. Fish Pathol.

[3] Liang Y, Liang B (2007). Spatial distribution of the protozoan parasite *Perkinsus* sp. found in the Manila clams *Ruditapes philippinarum* in China. World Aquaculture 2007 Abstract book.

[4] Ruano F, Batista FM, Arcangeli G (2015). Perkinsosis in the clams *Ruditapes decussatus* and *R. philippinarum* in the northeastern Atlantic and Mediterranean Sea: a review. J Invertebr Pathol.

[5] Choi KS, Park KI. Review on the protozoan parasite *Perkisus olseni* (Lester and Davis 1981) infection in Asian waters. In: Ishimatsu A, Lie HJ, editors. Coastal environmental and ecosystem issues of the East China Sea: TERRAPUB and Nagasaki University Nagasaki; 2010. p. 269–81.

[6] Park KI, Tsutsumi H, Hong JS, Choi KS. Pathology survey of the short-neck clam *Ruditapes philippinarum* occurring on sandy tidal flats along the coast of Ariake Bay, Kyushu, Japan. J Invertebr Pathol. 2008;99:212–9. 10.1016/j.jip.2008.06.004 18602398

[7] Lee MK, Cho BY, Lee SJ, Kang JY, Jeong HD, Huh SH (2008). Histopathological lesions of Manila clam, *Tapes philippinarum*, from Hadong and Namhae coastal areas of Korea. Aquaculture..

[8] Park KI, Figueras A, Choi KS (2006). Application of enzyme-linked immunosorbent assay (ELISA) for the study of reproduction in the Manila clam *Ruditapes philippinarum*: (Mollusca: Bivalvia): II. Impacts of *Perkinsus olseni* on clam reproduction. Aquaculture..

[9] Nam KW, Jeung HD, Song JH, Park KH, Choi KS, Park KI (2018). High parasite burden increase the surfacing and mortality of the manila clam (*Ruditapes philippinarum*) in intertidal sandy mudflats on the west coast of Korea during hot summer. Parasites Vectors.

[10] Waki T, Takahashi M, Eki T, Hiasa M, Umeda K, Karakawa N (2018). Impact of *Perkinsus olseni* infection on a wild population of Manila clam *Ruditapes philippinarum* in Ariake Bay, Japan. J. Invertebr. Pathol.

[11] Park KI, Park JK, Lee J, Choi KS (2005). Use of molecular markers for species identification of Korean *Perkinsus* sp. isolated from Manila clam, *Ruditapes philippinarum* in Korean water. Dis Aquat Org.

[12] Wu SQ, Wang CX, Lin XM, Wang ZX, Li XF, Liu J (2011). Infection prevalence and phylogenetic analysis of *Perkinsus olseni* in *Ruditapes philippinarum* from East China. Dis Aquat Org.

[13] Dungan CF, Reece KS (2006). *In Vitro* propagation of two *Perkinsus* spp. parasites from Japanese Manila clams *Venerupis philippinarum* and description of *Perkinsus honshuensis* n. sp. J Eukaryot Microbiol.

[14] Kang HS, Yang HS, Reece KS, Hong HK, Park KI, Choi KS (2016). First report of *Perkinsus honshuensis* in the variegated carpet shell clam *Ruditapes variegatus* in Korea. Dis Aquat Org.

[15] Kang HS, Yang HS, Reece K, Cho YG, Lee HM, Kim CW (2017). Survey on *Perkinsus* species in Manila clam *Ruditapes philippinarum* in Korean waters using species-specific PCR. Fish Pathol..

[16] Auzoux-Bordenave S, Vigario AM, Ruano F, Domart-Coulon I, Doumenc D (1995). *In vitro* sporulation of the clam pathogen *Perkinsus atlanticus* (Apicomplexa, Perkinsea) under various environmental conditions. J Shellfish Res.

[17] Perkins FO (1996). The structure of *Perkinsus marinus* (Mackin, Owen, and collier, 1950) Levine, 1978, with comments on the taxonomy and phylogeny of *Perkinsus* spp. J Shellfish Res.

[18] Choi KS, Park KI, Cho M, Soudant P (2005). Diagnosis, pathology, and taxonomy of *Perkinsus* sp. isolated from the Manila clam *Ruditapes philippinarum* in Korea. Korean J. Aquacult.

[19] Ordás MC, Novoa B, Figueras A (1999). Phagocytosis inhibition of clam and mussel haemocytes by *Perkinsus atlanticus* secretion products. Fish Shellfish Immunol.

[20] Pretto T, Zambon M, Civettini M, Caburlotto G, Boffo L, Rossetti E (2014). Massive mortality in Manila clams (*Ruditapes philippinarum*) farmed in the lagoon of Venice, caused by *Perkinsus olseni*. Bull Eur Assoc Fish Pathol.

[21] Chu FLE, Greene KH (1989). Effect of temperature and salinity on *in vitro* culture of the oyster pathogen, *Perkinsus marinus* (Apicomplexa: Perkinsea). J Invertebr Pathol.

[22] Casas SM, Villalba A, Reece KS (2002). Study of perkinsosis in the carpet shell clam *Tapes decussatus* in Galicia (NW Spain). I. Identification of the aetiological agent and in vitro modulation of zoosporulation by temperature and salinity. Dis Aquat. Org.

[23] Ray SM (1952). A culture technique for the diagnosis of infection with *Dermocystidium marinum* Mackin, Owen, and collier in oysters. Science..

[24] Ray SM (1954). Biological studies of *Dermocystidium marinum*. Rice Inst Pamph.

[25] Mackin JG (1962). Oyster disease caused by *Dermocystidium marinum* and other microorganisms in Louisiana. Publ Inst Mar Sci Univ Texas.

[26] Park KI, Yang HS, Kang HS, Cho M, Park KJ, Choi KS (2010). Isolation and identification of *Perkinsus olseni* from feces and marine sediment using immunological and molecular techniques. J Invertebr Pathol.

[27] Dungan CF, Reece KS, Moss JA, Hamilton RM, Diggles BK (2007). *Perkinsus olseni* in vitro isolates from the New Zealand clam *Austrovenus stutchburyi*. J Eukaryot Microbiol.

[28] Ordas MC, Figueras A (1998). *In vitro* culture of *Perkinsus atlanticus*, a parasite of the carpet shell clam *Ruditapes decussatus*. Dis Aquat Org.

[29] Perkins FO, Menzel RW (1967). Ultrastructures of sporulation in the oyster pathogen *Dermocystidium marinum*. Proc Natl Shellfish Assoc.

[30] Leander BS, Hoppernrath M (2007). Ultrastructure of novel tube-forming, intracellular parasite of dinoflagellates: *Parvilucifera prorocentri* sp. Nov. (Alveolata, Myzozoa). Eur J Protistol.

[31] Hoppenrath M, Leander BS (2009). Molecular phylogeny of *Parvilucifera prorocentri* (Alveolata, Myzozoa): insights into Perkinsid character evolution. J Eukaryot Microbiol.

[32] Sunila I, Hamilton RM, Dungan CF (2001). Ultrastructural characteristics of the in vitro cell cycle of the protozoan pathogen of oysters, *Perkinsus marinus*. J Eukaryot Microbiol.

[33] Gauthier JD, Vasta GR (1995). *In vitro* culture of the eastern oyster parasite *Perkinsus marinus:* optimization of the methodology. J Invertebr Pathol.

[34] Quarmby LM (2004). Cellular deflagellation. Int Rev Cytol.

[35] Harris EH (1989). Chlamydomonas sourcebook: A comprehensive guide to biology and laboratory use.

[36] McNally FJ, Vale RD (1993). Identification of katanin, an ATPase that severs and disassembles stable microtubules. Cell..

[37] Sanders MA, Salisbury JL (1994). Centrin plays an essential role in microtubule severing during flagellar excision in *Chlamydomonas reinhardtii*. J Cell Biol.

[38] Pereira-Neves A, Ribeiro KC, Benchimol M (2003). Pseudocysts in trichomonasds- new insights. Protist..

[39] Quarmby LM, Parker JDK (2005). Cilia and the cell cycle?. J Cell Biol.

[40] Midlej V, Benchimol M (2009). *Giardia lamblia* behavior during encystment: how morphological changes in shape occur. Parasitol Int.

[41] Bastin P, Pullen TJ, Moreira-Leite FF, Gull K (2000). Inside and outside of the trypanosome flagellum: a multifunctional organelle. Microbes Infect.

[42] Wilson-Ormond EA, Powell EN, Choi KS, Song J (1993). *Perkinsus marinus* assay. In: Lauenstein GG, Cantillo AY, editors. Sampling and analytical methods of the National Status and trends program National Benthic Surveillance and mussel watch projects, 1984-1992. Volume II. Comprehensive description of complementary measurements. NOAA Technical Memorandum NOS ORCA.

[43] Park K-I, Park Y-M, Lee (2002). Development of a PCR assay for detection of the protozoan parasite *Perkinsus*. Korean J Environ Biol.

