# Correction to: Scanning electron microscopic observation of the in vitro cultured protozoan, Perkinsus olseni, isolated from the Manila clam, Ruditapes philippinarum

**DOI:** 10.1186/s12866-020-01978-2

**Published:** 2020-09-18

**Authors:** Dinesh Gajamange, Seung-Hyeon Kim, Kwang-Sik Choi, Carlos Azevedo, Kyung-Il Park

**Affiliations:** 1 Department of Aquatic Life Medicine, College of Ocean Science and Technology, Kunsan National University, 558 Daehakno, Gunsan, 54150 Republic of Korea; 2 Present address: The Open University of Sri Lanka, Regional Centre, Matara, Sri Lanka; 3 School of Marine Biomedical Sciences, College of Ocean Sciences, Jeju National University, 102 Jejudaehakno, Jeju, 63243 Republic of Korea; 4 Laboratory of Cell Biology, Institute of Biomedical Sciences, University of Porto, Porto, Portugal

**Correction to: BMC Microbiol (2020) 20:238**

**https://doi.org/10.1186/s12866-020-01926-0**

Following publication of the original article [1], we were notified of an error in Fig. 2. Correct Fig. 2 is presented below, providing clearer morphological information about the pathogen.

**Fig. 2:**
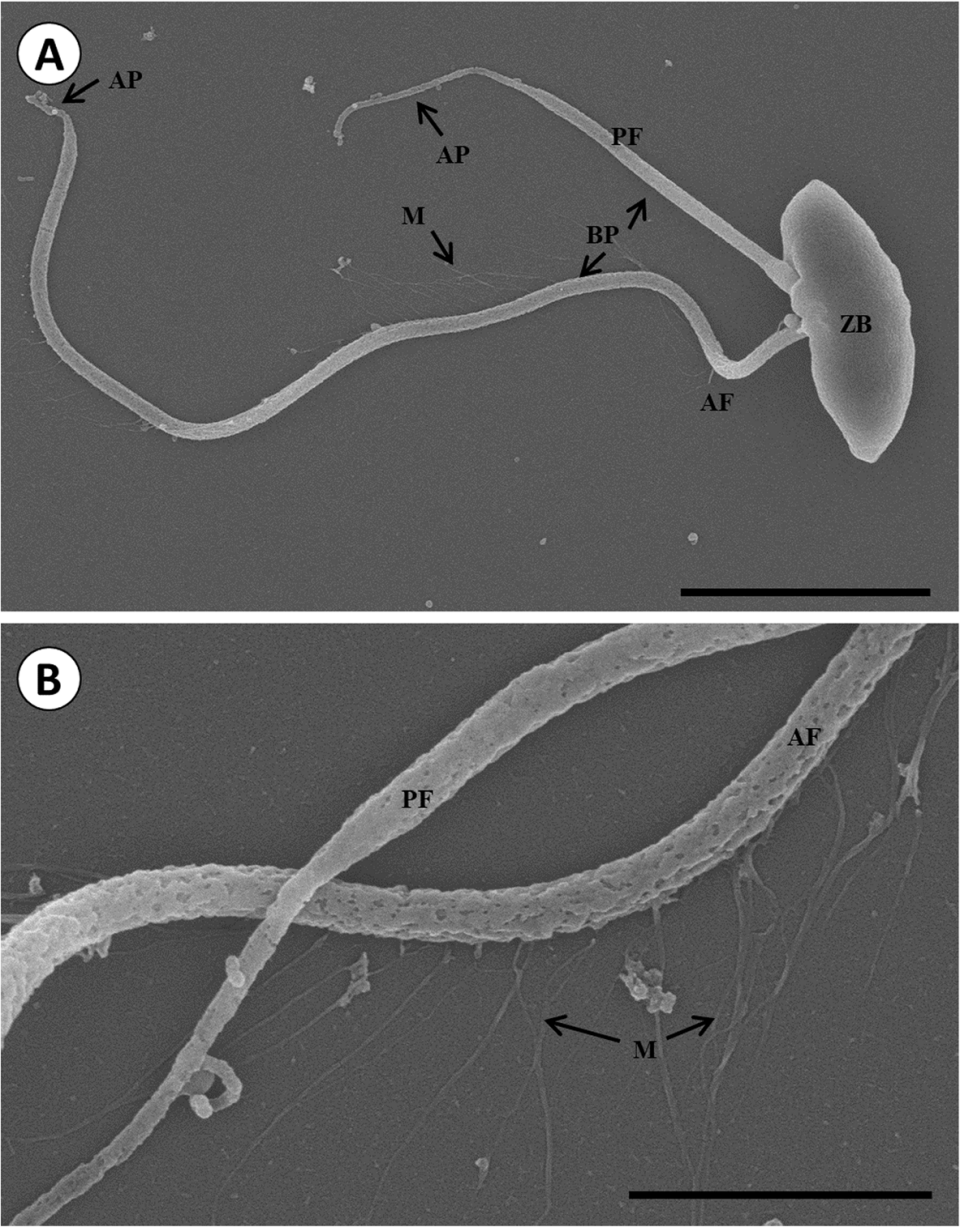
Scanning electron micrograph of *P. olseni* zoospore (**a**) and tinsel (mastigonemes, M) (**b**). A zoospore with the anterior flagellum (AF) and posterior flagellum (PF). The flagellum is rooted in the zoospore body (ZB). The flagellum is divided into two parts; the basal portion (BP) and an apical portion (AP). Unilateral array of tinsels on the anterior flagellum (M). Scale bar = 2 μm (**a**), Scale bar = 1 μm (**b**)
